# Pretreatment neutrophil percentage-to-albumin ratio for prognostic assessment in locally advanced nasopharyngeal carcinoma

**DOI:** 10.17305/bb.2026.14023

**Published:** 2026-04-24

**Authors:** Yanming Hu, Luting Chen, Jie Ling, Sujuan Zhang, Yuhua Feng, Tao Hou, Yangchun Xie

**Affiliations:** 1 Department of Oncology, The Second Xiangya Hospital, Central South University, Changsha, Hunan, China

**Keywords:** NPAR, LA-NPC, radiotherapy, nomogram, prognosis

## Abstract

Prognosis in locally advanced nasopharyngeal carcinoma (NPC) is influenced not only by tumor burden but also by systemic inflammatory and nutritional status. This study aimed to evaluate the prognostic value of the pretreatment neutrophil percentage-to-albumin ratio (NPAR) in locally advanced NPC and to determine whether its incorporation could enhance risk stratification beyond conventional staging variables. In this retrospective single-center cohort study, we analyzed 804 patients with stage II-IVa NPC who were treated with definitive radiotherapy, with or without concurrent chemotherapy. These patients were randomly assigned to a training cohort (*n* ═ 603) and a validation cohort (*n* ═ 201). The pretreatment NPAR was calculated from blood samples collected within one week prior to treatment initiation. Associations with overall survival (OS) and progression-free survival (PFS) were primarily assessed using restricted cubic spline Cox models, supplemented by secondary cutoff-based analyses. A prognostic nomogram was developed and internally validated through bootstrap resampling. Higher pretreatment NPAR was consistently associated with adverse outcomes in OS (adjusted hazard ratio [HR] 1.77, 95% confidence interval [CI] 0.97–3.23; *P ═* 0.062) and PFS (adjusted HR 1.74, 95% CI 0.96–3.15; *P ═* 0.066), although these associations did not achieve conventional statistical significance in fully adjusted models. The nomogram incorporating both clinicopathological variables and NPAR demonstrated superior discrimination compared to tumor-node-metastasis staging alone, with optimism-corrected C-indices of 0.625 for OS and 0.617 for PFS. In comparative analyses, NPAR and the neutrophil-to-lymphocyte ratio exhibited similar prognostic performance. In conclusion, pretreatment NPAR is associated with adverse outcomes in locally advanced NPC and may modestly enhance model-based risk stratification. However, its clinical utility should be interpreted with caution and requires external validation.

## Introduction

Nasopharyngeal carcinoma (NPC) poses a significant health challenge, particularly in high-incidence areas such as Southeast Asia and southern China [1, 2]. Despite advancements in treatment modalities, predicting prognosis remains difficult due to the complex interplay of various factors affecting disease progression and patient outcomes [3–5].

Emerging evidence suggests that prognosis in locally advanced NPC is influenced not only by anatomical tumor burden but also by the host’s inflammatory and nutritional status [6, 7]. Several blood-based markers have demonstrated prognostic relevance in NPC, including indices related to albumin and inflammation [8]. While NPC is strongly associated with Epstein-Barr virus-induced immune dysregulation, neutrophil-dominant systemic inflammation and nutritional depletion are also significant factors in disease progression and treatment tolerance [9, 10].

The neutrophil percentage-to-albumin ratio (NPAR) serves as a straightforward composite marker reflecting both inflammatory activation and nutritional reserve [11–16]. In this study, we evaluated pretreatment NPAR in a retrospective cohort of patients with locally advanced NPC undergoing definitive radiotherapy. We assessed its association with survival outcomes using both continuous and cutoff-based approaches and examined whether incorporating NPAR could enhance model-based risk stratification beyond conventional staging variables.

## Materials and methods

### Patient enrollment and study design

Patients diagnosed with NPC at the Second Xiangya Hospital, Central South University, between 2012 and 2019 were retrospectively screened for eligibility. Inclusion criteria were as follows: (1) age between 18 and 70 years; and (2) histologically confirmed nasopharyngeal squamous cell carcinoma staged II to IVa according to the American Joint Committee on Cancer (AJCC) 8th edition tumor-node-metastasis (TNM) classification. Treatment options included cisplatin-based concurrent chemoradiotherapy (CCRT) or radiotherapy with or without adjuvant chemotherapy (non-CCRT). Patients with a history of prior malignancy, previous exposure to radiotherapy or chemotherapy, or incomplete key clinical or outcome data were excluded from the final analytic dataset. Ethical clearance (approval No. LYEC2024-0321) was obtained from the Ethics Committee of the Second Xiangya Hospital, Central South University. Due to the retrospective nature of the study, informed consent was waived, and the study adhered to the Declaration of Helsinki.

### Treatment protocol

All participants underwent a comprehensive pretreatment evaluation, including medical history review, physical examination, endoscopic biopsy, laboratory testing, and cross-sectional imaging. Tissue samples for pathological analysis were obtained via nasopharyngoscopy, complemented by imaging examinations such as magnetic resonance imaging, computed tomography, emission computed tomography, and positron emission tomography as needed to assess regional lymph node and distant metastatic status. TNM staging, according to the 8th edition of the AJCC staging system, was independently evaluated by three experienced radiologists. All patients received intensity-modulated radiation therapy (IMRT), with prescribed doses of 70-74 Gy to the planning gross tumor volume (pGTV), 66-70 Gy to the planning gross tumor volume of nodal disease (pGTVnd), 60-62 Gy to planning target volume 1 (PTV1), and 50-56 Gy to planning target volume 2 (PTV2), delivered in 30-33 fractions. Target delineation adhered to institutional NPC radiotherapy protocols consistent with contemporary international recommendations. Cisplatin-based or nedaplatin-based chemotherapy was administered every three weeks for up to three cycles.

### Follow-up

Overall survival (OS) was designated as the primary endpoint, defined as the time from diagnosis to death from any cause or last follow-up. Progression-free survival (PFS), distant metastasis-free survival (DMFS), and recurrence-free survival (RFS) were evaluated as secondary or exploratory endpoints. PFS was defined as the time from diagnosis to disease progression, death, or last follow-up, while DMFS was defined as the time from diagnosis to distant metastasis or death, and RFS was defined as the time from diagnosis to locoregional recurrence. Follow-up was scheduled every three months for the first three years, every six months during years four and five, and annually thereafter. The final follow-up date was November 20, 2023. The median follow-up duration, calculated using the reverse Kaplan-Meier method, was 69.3 months (95% confidence interval [CI] 67.1–72.2).

**Table 1:** Comparison of demographic and clinicopathologic characteristics between the training and validation cohorts

**Characteristics**	**Total (*n* ═ 804)**	**Training set (*n* ═ 603)**	**Validation set (*n* ═ 201)**	***P* value**
Age, mean ± SD	48.23 ± 10.69	48.35 ± 10.83	47.89 ± 10.28	0.590
Sex				0.318
Female	224 (27.9%)	162 (26.9%)	62 (30.8%)	
Male	580 (72.1%)	441 (73.1%)	139 (69.2%)	
T stage				0.392
T1-2	413 (51.4%)	304 (50.4%)	109 (54.2%)	
T3-4	391 (48.6%)	299 (49.6%)	92 (45.8%)	
N stage				0.141
N0-1	195 (24.3%)	138 (22.9%)	57 (28.4%)	
N2-3	609 (75.7%)	465 (77.1%)	144 (71.6%)	
TNM stage				0.557
I-II	112 (13.9%)	81 (13.4%)	31 (15.4%)	
III-IV	692 (86.1%)	522 (86.6%)	170 (84.6%)	
ECOG				0.431
0	477 (59.3%)	363 (60.2%)	114 (56.7%)	
1-2	327 (40.7%)	240 (39.8%)	87 (43.3%)	
Treatment				0.632
non-CCRT	545 (67.8%)	412 (68.3%)	133 (66.2%)	
CCRT	259 (32.2%)	191 (31.7%)	68 (33.8%)	
BMI, mean ± SD	23.35 ± 3.13	23.30 ± 3.12	23.49 ± 3.17	0.480
Drinking				0.118
No	509 (63.3%)	372 (61.7%)	137 (68.2%)	
Yes	295 (36.7%)	231 (38.3%)	64 (31.8%)	
Smoking				0.402
No	629 (78.2%)	467 (77.4%)	162 (80.6%)	
Yes	175 (21.8%)	136 (22.6%)	39 (19.4%)	
Family history				0.251
No	783 (97.4%)	590 (97.8%)	193 (96.0%)	
Yes	21 (2.6%)	13 (2.2%)	8 (4.0%)	
NPAR, mean ± SD	1.53 ± 0.25	1.53 ± 0.26	1.51 ± 0.24	0.321

Data are presented as mean ± SD or *n* (%). *P* values were calculated using the independent-samples *t*-test or chi-square test, as appropriate. **Abbreviations:** BMI, body mass index; CCRT, concurrent chemoradiotherapy; ECOG, Eastern Cooperative Oncology Group performance status; n, number; NPAR, neutrophil percentage-to-albumin ratio; SD, standard deviation; TNM, tumor-node-metastasis.

### Statistical analysis

Patients were randomly divided into training and validation cohorts at a 3:1 ratio. Pretreatment blood samples were collected within one week prior to the initiation of radiotherapy or chemoradiotherapy. NPAR was calculated as the neutrophil percentage (%) divided by serum albumin concentration (g/L). The neutrophil-to-lymphocyte ratio (NLR) was calculated using absolute neutrophil and lymphocyte counts. Continuous variables were assessed for normality using the Shapiro-Wilk test and compared using Student’s *t*-test or the Mann–Whitney *U* test as appropriate. Categorical variables were compared using the chi-square test or Fisher’s exact test. To minimize overreliance on dichotomization, the association between NPAR and survival outcomes was initially evaluated as a continuous variable using Cox proportional hazards models with restricted cubic splines. An optimal NPAR cutoff was subsequently determined using X-tile software in the training cohort based on OS and then applied unchanged to the validation cohort. Multivariable Cox regression models were constructed using prespecified clinically relevant covariates, including age, Eastern Cooperative Oncology Group (ECOG) performance status, and disease-burden variables. The final OS model retained age, ECOG performance status, T stage, and N stage. The final PFS model retained age, T stage, and N stage. As TNM stage is derived from T stage and N stage, it was not included simultaneously with T stage and N stage in the final multivariable model to avoid collinearity; instead, TNM stage was evaluated as a comparator model. The proportional hazards assumption was assessed using Schoenfeld residuals. A prognostic nomogram was developed based on the final multivariable model. Model discrimination was evaluated using Harrell’s concordance index (C-index) and time-dependent receiver operating characteristic (ROC) analysis for censored survival data at three, five, and seven years, with 95% CIs. Internal validation and optimism correction were performed using bootstrap resampling. Calibration was assessed using calibration plots, calibration intercepts, calibration slopes, and Brier scores. Comparative analyses were also conducted for the available inflammation-related marker NLR. C-reactive protein (CRP)-derived indices, including the C-reactive protein-to-albumin ratio (CAR), Glasgow Prognostic Score (GPS), and modified Glasgow Prognostic Score (mGPS), could not be calculated because of the absence of CRP data in the retrospective dataset.

### Ethical statement

This study was reviewed and approved by the Ethics Committee of the Second Xiangya Hospital of Central South University, with approval number: LYEC2024-0321, dated August 28, 2024.

## Results

### Comparative analysis: Baseline characteristics of patient cohorts

A total of 804 patients were included in the final analysis, consisting of 603 patients in the training cohort and 201 patients in the validation cohort. Baseline clinicopathological characteristics were generally balanced between the two cohorts (Table 1). The mean age at diagnosis was 48.23 ± 10.69 years, with 580 patients (72.1%) being male. In the overall cohort, 545 patients (67.8%) received non-CCRT treatment, while 259 patients (32.2%) underwent CCRT. According to the AJCC 8th edition staging system, 692 patients (86.1%) had stage III-IV disease, and 609 patients (75.7%) had N2-3 disease. The mean NPAR was 1.53 ± 0.25.

As a secondary stratified analysis, the X-tile-derived cutoff value of 1.8 was established in the training cohort and subsequently applied unchanged to the validation cohort (Figure S1). Baseline characteristics stratified by the resulting low- and high-NPAR groups in the training and validation cohorts are summarized in Table S1. To mitigate the risk of data-driven over-interpretation, the primary inferential analyses were based on continuous modeling of NPAR rather than solely on dichotomization.

**Figure 1.**
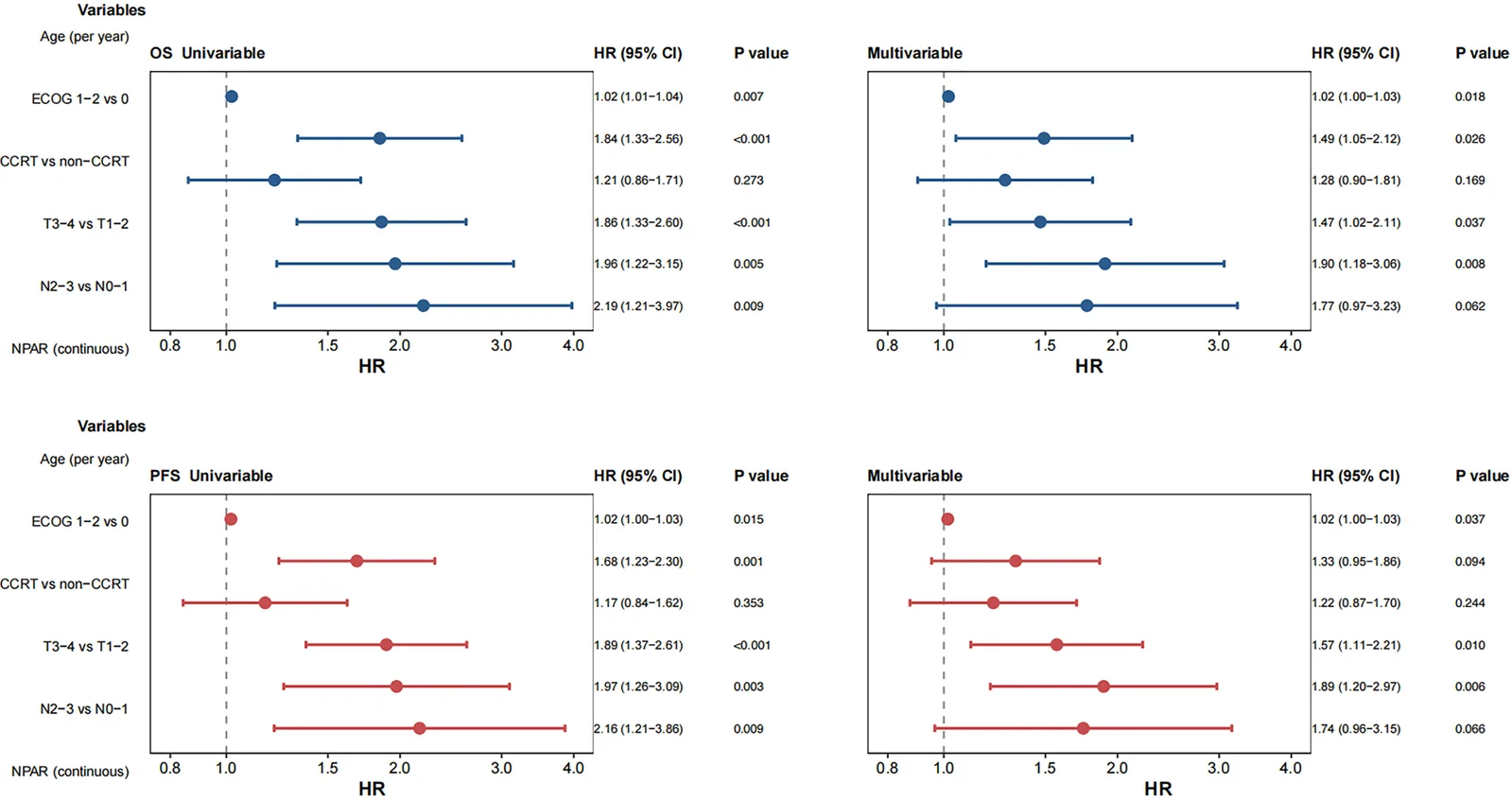
**Univariable and multivariable Cox analyses for OS and progression-free survival in the training cohort.** The forest plots illustrate hazard ratios with 95% confidence intervals for the predefined final Cox models. The final OS model retained variables including age, ECOG performance status, T stage, and N stage. The final PFS model included age, T stage, and N stage. The continuous NPAR was incorporated as the primary exposure variable in both models. **Abbreviations:** ECOG, Eastern Cooperative Oncology Group; N, nodal stage; NPAR, neutrophil percentage-to-albumin ratio; OS, overall survival; PFS, progression-free survival; T, tumor stage.

### Survival and prognostic factors analysis

When modeled as a continuous variable, higher pretreatment NPAR was consistently associated with poorer OS and PFS. In the prespecified multivariable Cox model, the adjusted hazard ratio (HR) for NPAR was 1.77 (95% CI 0.97-3.23; *P ═* 0.062) for OS and 1.74 (95% CI 0.96-3.15; *P ═* 0.066) for PFS. Other variables retained in the final model included age, ECOG performance status, T stage, and N stage for OS, as well as age, T stage, and N stage for PFS. The corresponding univariable and multivariable effect estimates are summarized in Figure 1. In secondary cutoff-based Kaplan-Meier analyses, patients with higher NPAR values exhibited less favorable survival outcomes for both OS and PFS (Figure 2). Continuous risk patterns are further illustrated by the restricted cubic spline curves in Figure 3. Additional categorical survival curves are presented as Figures S2−S7.

### Nomogram development and validation

The nomogram demonstrated superior discrimination compared to TNM staging alone within the same internal validation framework. For OS, the nomogram achieved C-indices of 0.643 (95% CI 0.597-0.679) in the training cohort and 0.697 (95% CI 0.619-0.765) in the validation cohort, compared to 0.540 (95% CI 0.514-0.559) and 0.571 (95% CI 0.540-0.597) for TNM stage alone. For PFS, the corresponding C-indices were 0.635 (95% CI 0.589-0.670) and 0.685 (95% CI 0.618-0.745) for the nomogram, versus 0.537 (95% CI 0.512-0.557) and 0.564 (95% CI 0.534-0.595) for TNM stage alone. The optimism-corrected C-index derived from bootstrap resampling was 0.625 for OS and 0.617 for PFS, which are slightly lower than the apparent C-indices (0.643/0.697 for OS; 0.635/0.685 for PFS), as expected due to corrections for overfitting. Calibration and discrimination results for the revised nomogram are depicted in Figures 4-6.

### Assessment of model performance

Time-dependent ROC analysis further validated the performance of the nomogram (Figure 5). For OS, the 3-, 5-, and 7-year area under the curves (AUCs) were 0.646, 0.656, and 0.717 in the training cohort, and 0.728, 0.705, and 0.717 in the validation cohort. For PFS, the corresponding AUCs were 0.653, 0.648, and 0.702 in the training cohort, and 0.710, 0.715, and 0.719 in the validation cohort. For the OS nomogram in the validation cohort, the calibration intercept was −0.02 (95% CI −0.11 to 0.07), the calibration slope was 0.96 (95% CI 0.85 to 1.07), and the Brier score was 0.18. For the PFS nomogram, the corresponding values were: intercept −0.03 (95% CI −0.12 to 0.06), slope 0.94 (95% CI 0.82 to 1.06), and Brier score 0.20. These results indicate robust calibration.

**Figure 2.**
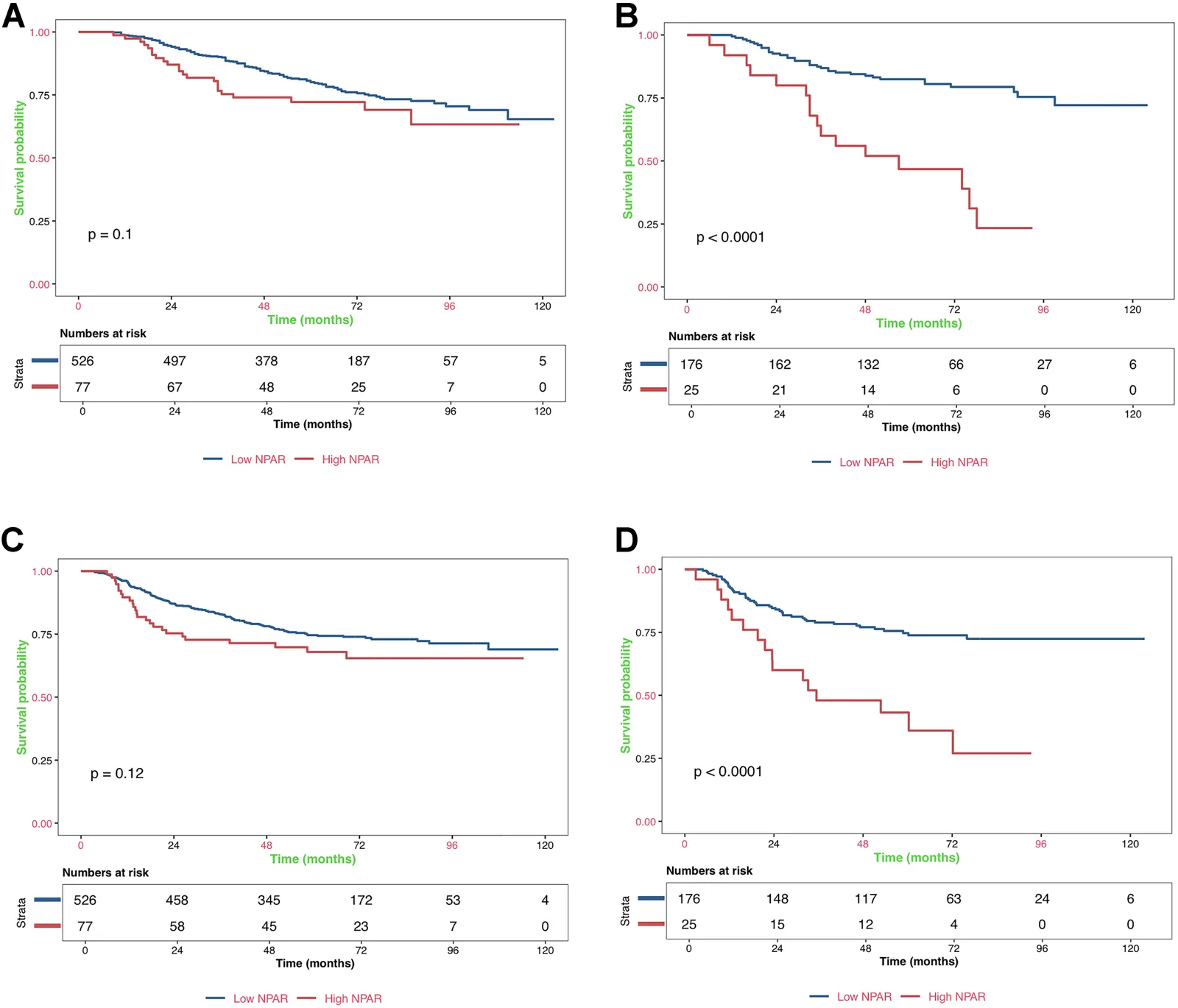
**Kaplan–Meier survival curves for OS and progression-free survival according to the pretreatment neutrophil percentage-to-albumin ratio in the training and validation cohorts.** Patients were stratified into low and high NPAR groups using the X-tile-derived cutoff established in the training cohort and applied unchanged to the validation cohort. (A) OS in the training cohort. (B) OS in the validation cohort. (C) PFS in the training cohort. (D) PFS in the validation cohort. Higher NPAR values were associated with less favorable survival patterns for both endpoints, with modest curve separation in the training cohort and more pronounced separation in the validation cohort. *P* values shown in the panels were derived from log-rank tests, and numbers at risk are presented below each plot. **Abbreviations:** NPAR, neutrophil percentage-to-albumin ratio; OS, overall survival; PFS, progression-free survival.

**Figure 3.**
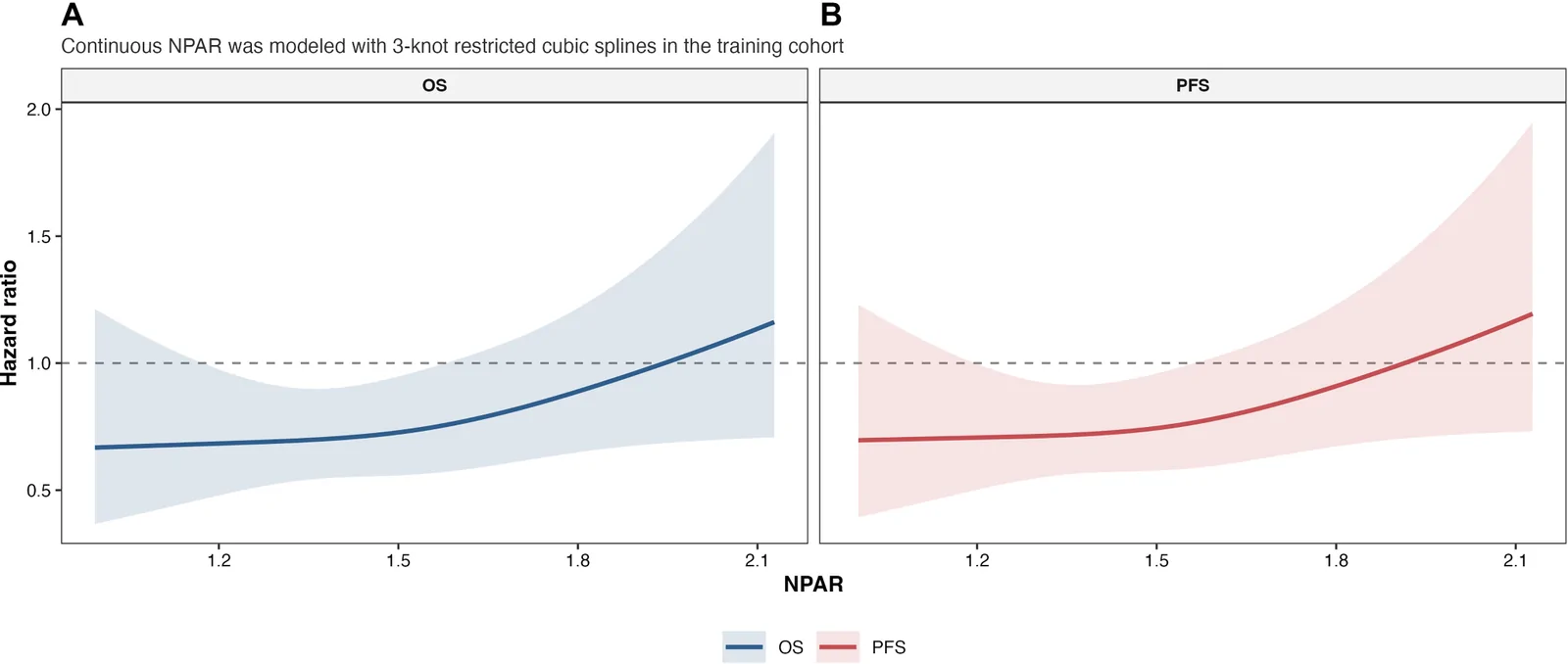
**Restricted cubic spline analysis of continuous NPAR in the training cohort.** The restricted cubic spline curves illustrate the adjusted relationship between continuous pretreatment NPAR and the hazard of OS (A) and PFS (B). Solid lines represent adjusted hazard ratios, while shaded areas denote 95% confidence intervals. **Abbreviations:** NPAR, neutrophil percentage-to-albumin ratio; OS, overall survival; PFS, progression-free survival.

**Figure 4.**
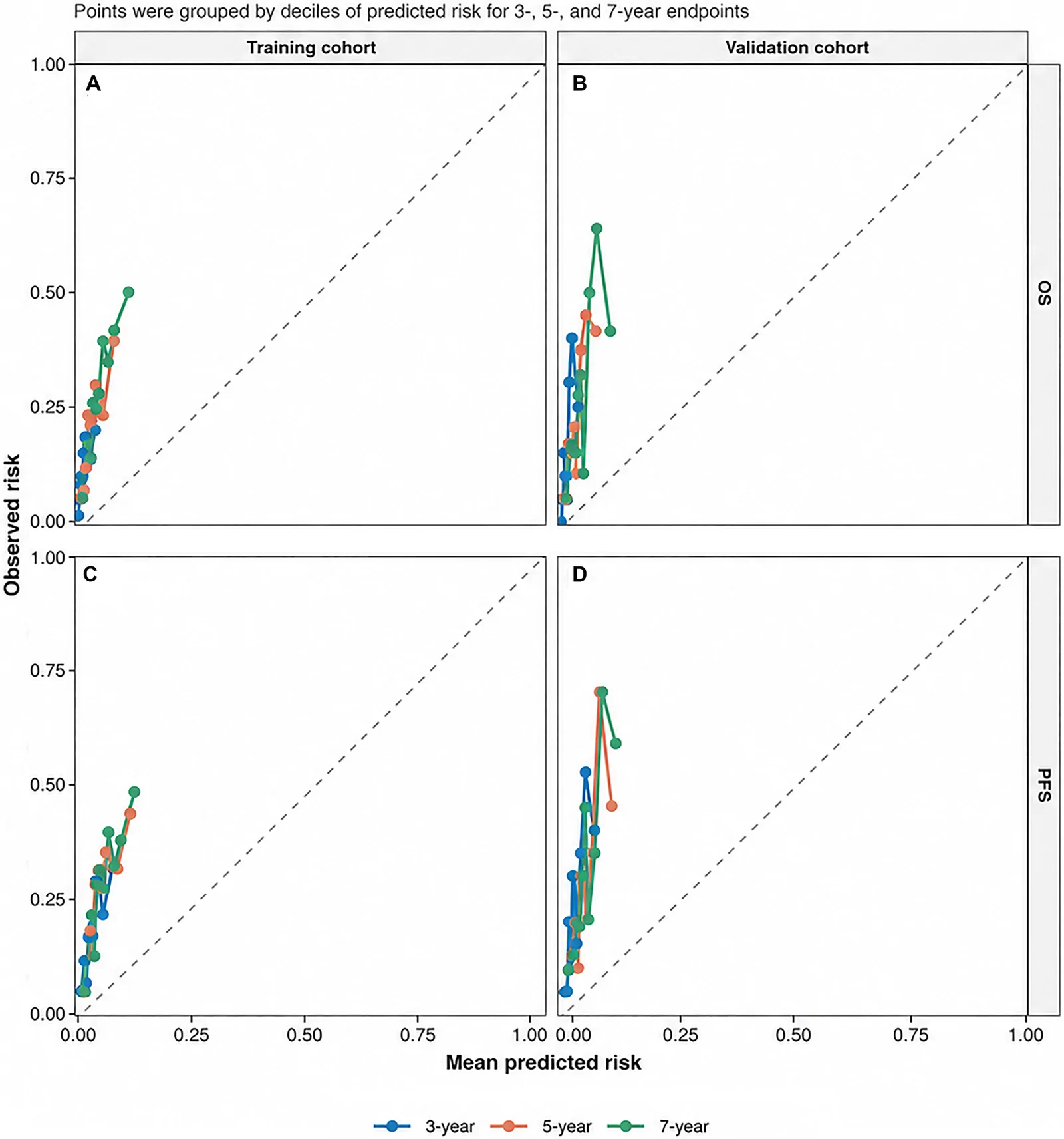
**Calibration plots of the revised nomogram for OS and progression-free survival in the training and validation cohorts.** The plots compare mean predicted risk with observed risk for the 3-year, 5-year, and 7-year predictions. (A) OS in the training cohort. (B) OS in the validation cohort. (C) PFS in the training cohort. (D) Progression-free survival in the validation cohort. Colored points and connecting lines represent calibration at the three prediction time points, and the dashed 45∘ line indicates perfect agreement between predicted and observed risk. **Abbreviations:** OS, overall survival; PFS, progression-free survival.

**Figure 5.**
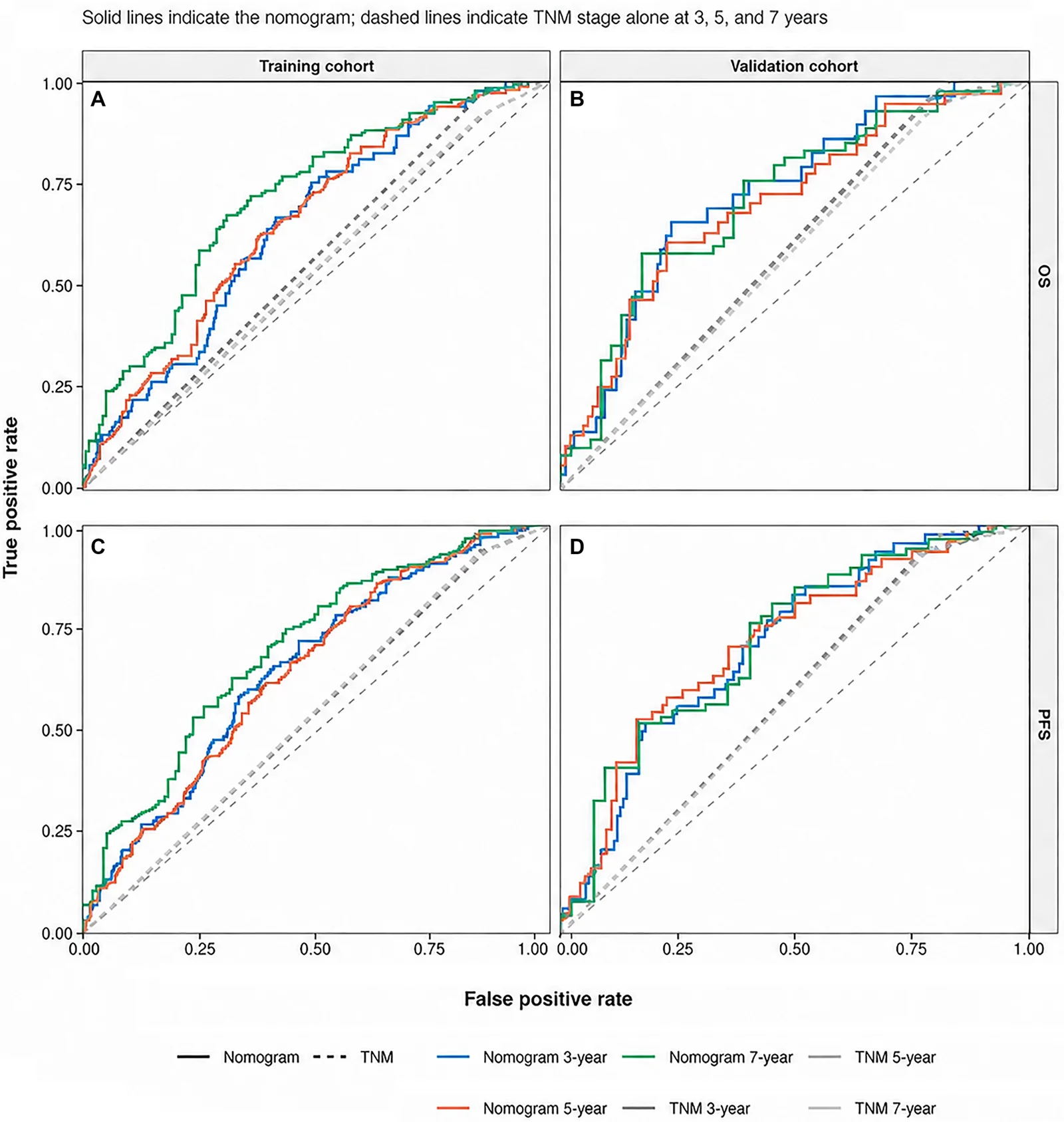
**Time-dependent receiver operating characteristic curves for the nomogram and TNM stage.** This figure illustrates time-dependent ROC curves for OS and PFS in both the training and validation cohorts at 3, 5, and 7 years. The solid colored lines represent the nomogram, while the dashed grey lines indicate the TNM stage alone. **Abbreviations:** OS, overall survival; PFS, progression-free survival; ROC, receiver operating characteristic; TNM, tumor-node-metastasis.

**Figure 6.**
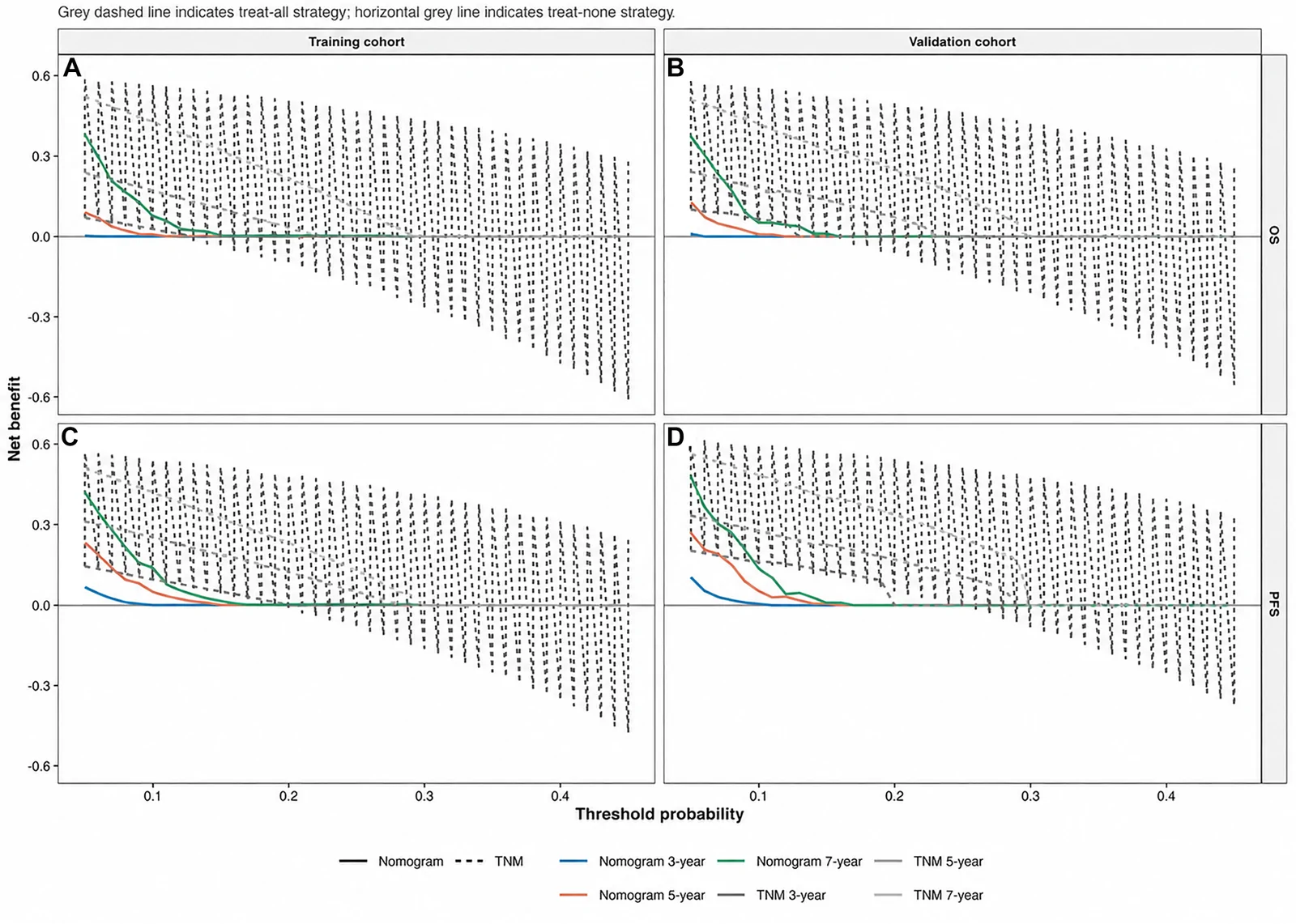
**Decision curve analysis comparing the revised nomogram with TNM stage for OS and progression-free survival in the training and validation cohorts.** Net benefit was plotted across clinically relevant threshold probabilities for the 3-, 5-, and 7-year predictions. (A) OS in the training cohort. (B) OS in the validation cohort. (C) PFS in the training cohort. (D) PFS in the validation cohort. Colored solid lines represent the nomogram, whereas gray dashed lines represent TNM stage alone. The sloping dashed reference curve indicates the treat-all strategy, and the horizontal line at a net benefit of zero indicates the treat-none strategy. Across a range of lower threshold probabilities, the nomogram generally showed greater net benefit than TNM stage alone for both endpoints in the training and validation cohorts. **Abbreviations:** OS, overall survival; PFS, progression-free survival; TNM, tumor-node-metastasis.

### Comparison of prognostic accuracy for OS and PFS with NLR

Due to the unavailability of CRP in the retrospective dataset, the CAR and GPS/mGPS could not be reliably calculated. Consequently, a direct comparison between NPAR and NLR, which was available for the entire cohort, was performed. For OS, NPAR and NLR exhibited similar prognostic performance, with adjusted HRs per standardized increase of 1.16 (95% CI 0.99-1.35; *P ═* 0.062) for NPAR and 1.13 (95% CI 1.00-1.27; *P ═* 0.057) for NLR. For PFS, NLR demonstrated a slightly stronger adjusted association than NPAR, with adjusted HRs of 1.15 (95% CI 0.99-1.35; *P ═* 0.066) for NPAR and 1.15 (95% CI 1.03-1.30; *P ═* 0.015) for NLR. These comparative results are summarized in Figure S8. The patient selection process and allocation of the final analytic cohort into the training and validation cohorts are shown in Figure S9.

## Discussion

In this retrospective cohort of patients with locally advanced NPC treated with definitive radiotherapy, pretreatment NPAR consistently demonstrated an adverse association with clinical outcomes and contributed significantly to model-based risk stratification. Notably, the prognostic value of NPAR was primarily assessed using continuous modeling rather than relying exclusively on a data-driven cutoff, thereby minimizing the risk of overinterpretation due to dichotomization. Although the adjusted continuous association did not achieve conventional statistical significance in the fully prespecified model, the incorporation of NPAR into the nomogram enhanced discrimination compared to TNM staging alone.

The biological relevance of NPAR in NPC warrants attention. While NPC is closely associated with Epstein-Barr virus-related immune dysregulation, rendering lymphocyte-related biomarkers particularly pertinent, neutrophil-mediated systemic inflammation and compromised nutritional status remain critical factors influencing tumor progression and treatment tolerance [17-19]. Consequently, NPAR may encapsulate a clinically meaningful inflammation-nutrition axis in this disease context.

We also compared NPAR with the available inflammatory marker NLR to better contextualize its relative utility. In our cohort, NPAR and NLR exhibited broadly similar prognostic performance for OS, whereas NLR demonstrated a somewhat stronger adjusted association with PFS. Given that CRP was not routinely available in this retrospective dataset, reliable comparisons with CAR or GPS/mGPS were not feasible, and this limitation is explicitly acknowledged.

The revised nomogram should thus be interpreted as a risk-stratification tool that modestly improves prognostic discrimination beyond TNM staging alone, rather than as conclusive evidence that NPAR is universally superior to other inflammatory biomarkers.

Several limitations should be noted. First, this was a retrospective single-center study and is therefore subject to selection bias and residual confounding. Second, the current validation remains internal despite bootstrap optimism correction, and external validation is warranted. Third, the unavailability of CRP in the retrospective dataset precluded comparisons with CRP-derived indices such as CAR and GPS/mGPS.

## Conclusion

In conclusion, pretreatment NPAR demonstrated a consistent adverse association with outcomes in locally advanced NPC and enhanced nomogram-based risk stratification beyond TNM staging alone. Its clinical utility should be interpreted with caution and validated in prospective, externally validated studies.

## Supplemental data

Supplemental data are available at the following link: https://www.bjbms.org/ojs/index.php/bjbms/article/view/14023/4184.
